# Comparative performance of WANTAI ELISA for total immunoglobulin to receptor binding protein and an ELISA for IgG to spike protein in detecting SARS-CoV-2 antibodies in Kenyan populations

**DOI:** 10.1016/j.jcv.2021.105061

**Published:** 2022-01

**Authors:** James Nyagwange, Bernadette Kutima, Kennedy Mwai, Henry K. Karanja, John N. Gitonga, Daisy Mugo, Sophie Uyoga, James Tuju, Lynette I. Ochola-Oyier, Francis Ndungu, Philip Bejon, Ambrose Agweyu, Ifedayo M.O. Adetifa, J.Anthony G. Scott, George M. Warimwe

**Affiliations:** a KEMRI-Wellcome Trust Research Programme, PO Box 230, Kilifi, Kenya; b Department of Infectious Diseases Epidemiology, London School of Hygiene and Tropical Medicine, WC1E 7HT, Keppel Street, London, United Kingdom; c Nuffield Department of Medicine, Oxford University, OX3 7BN, Oxford, United Kingdom; d School of Public Health, Faculty of Health Sciences, University of the Witwatersrand, 27 St Andrews Road, Parktown 2193, Johannesburg, South Africa

**Keywords:** SARS-CoV-2, Immunoassay, IgG, Total immunoglobulin, Serology

## Abstract

Many SARS-CoV-2 antibody detection assays have been developed but their differential performance is not well described. In this study we compared an in-house (KWTRP) ELISA which has been used extensively to estimate seroprevalence in the Kenyan population with WANTAI, an ELISA which has been approved for widespread use by the WHO. Using a wide variety of sample sets including pre-pandemic samples (negative gold standard), SARS-CoV-2 PCR positive samples (positive gold standard) and COVID-19 test samples from different periods (unknowns), we compared performance characteristics of the two assays. The overall concordance between WANTAI and KWTRP was 0.97 (95% CI, 0.95–0.98). For WANTAI and KWTRP, sensitivity was 0.95 (95% CI 0.90–0.98) and 0.93 (95% CI 0.87–0.96), respectively. Specificity for WANTAI was 0.98 (95% CI, 0.96–0.99) and 0.99 (95% CI 0.96–1.00) while KWTRP specificity was 0.99 (95% CI, 0.98–1.00) and 1.00 using pre-pandemic blood donors and pre-pandemic malaria cross-sectional survey samples respectively. Both assays show excellent characteristics to detect SARS-CoV-2 antibodies.

## Highlights

• Both assays discriminate SARS-CoV-2 seropositive and seronegative individuals.
• Both assays correctly estimate seroprevalence in a wide range of seroprevalences.
• WANTAI has slightly higher sensitivity than KWTRP.
• KWTRP has slightly higher specificity than WANTAI.

## 1 Introduction

In December 2019, a severe acute respiratory syndrome coronavirus 2 (SARS-CoV-2) emerged in Wuhan and rapidly spread around the globe causing a pandemic. SARS-CoV-2 is an enveloped positive sense RNA virus with the genome encoding structural and non-structural proteins in the 3′ and 5′ regions respectively [1]. The spike (S) and the nucleoprotein (N) structural proteins are targets of neutralizing antibodies and immunodominant proteins respectively making them attractive for designing serological diagnostic kits [1].

Although nucleic acid amplification tests (NAAT) remain reference standard for SARS-CoV-2 diagnosis, serological assays have become very useful tools for estimating viral transmission and seroprevalence especially in regions where there is limited NAAT coverage, like Kenya. Sero-surveys are also useful for quantifying cumulative incidence of infection and viral transmission in regions with significant asymptomatic infection. We developed an in-house (KWTRP) ELISA based on the whole trimeric spike protein and validated it extensively on local samples and international standards. The validation process included participation in a WHO-sponsored multi-laboratory study of SARS-CoV-2 antibody assays [2]. The assay results were consistent with majority of the test laboratories [2]. We have since used the KWTRP ELISA to estimate SARS-CoV-2 seroprevalence in several target groups [3], [4], [5].

In this study we aimed to do a performance comparison between KWTRP ELISA measuring SARS-CoV-2 IgG and WANTAI ELISA measuring total immunoglobulins (Table 1) using the same set of samples. WANTAI is a commercial microplate ELISA based on the receptor binding domain of the SARS-CoV-2 spike protein and widely used around the world [6] and recommended by WHO, which has supplied the kits to us and other countries enrolled in UNITY studies [7].

**Table 1:** Description of the compared antibody detection ELISAs.

ELISA	WANTAI [8]	KWTRP [3]
Recombinant labeled protein	Receptor binding domain	Full length Spike
Antibody detected	Total Immunoglobulin	Immunoglobulin G
Methodology	Sandwich ELISA	Indirect ELISA
Sample type and volume	Serum/plasma 100µl	Serum/plasma 1 µl
Turnaround time	1.5h	5.5h
Cut-off calculation basis	negative control (min 0.19)	negative control (<0.2)
Threshold	ratio; 1.	ratio; >2X S/NC
Reported sensitivity	0.944 (95%CI, 0.909–0.968)	0.927 (95% CI, 0.879–0.961)
Reported specificity	1.00 (95%CI, 0.988–1.00)	0.99 (95% CI 0.981–0.995)

ELISA: Enzyme linked Immunosorbent Assay.

S/NC ratio between value of the sample (S) and value of the negative control (NC).

## 2 Materials and methods

Detailed characteristics of both assays are presented in Table 1.

## 3 WANTAI ELISA

The WANTAI ELISA was performed using the manufacturer's protocol. Briefly, 50 µl of positive and negative calibrator and 100 µl of serum or plasma samples were added to the plates and incubated at 37 °C for 30 min. After washing 5 times with diluted wash buffer, 100μl of HRP-Conjugate was added into each well except the Blank and incubated at 37 °C for 30 min. After further 5 washes, 50μl of Chromogen Solution A and 50μl of Chromogen Solution B were added and the plate incubated at 37 °C for 15 min avoiding light, then 50μl of Stop Solution added and the absorbance read at 450 nm. The ratio of absorbance (A) to cut-off (C.O.) (A/C.O. <1) was interpreted as having no SARS-CoV-2 antibodies (negative) and ≥1 having SARS-CoV-2 antibodies (positive).

### 3.1 KWTRP ELISA

The procedure has been reported extensively before [3]. Briefly, Nunc MaxiSorp™ flat-bottom 96-well plates (ThermoFisherScientific) were coated with 2 µg/ml of whole trimeric spike protein at 37 °C for 1 h, washed 3 times in wash buffer (0.1% Tween 20 in 1X phosphate buffered saline) and blocked with Blocker™ Casein (ThermoFisherScientific) for 1 h at room temperature. Then heat-inactivated serum or plasma samples were diluted 1:800 in Blocker™ Casein, added to the plates and incubated for 2 h at room temperature. After 3 further washes, 100 µl horseradish peroxidase-conjugated goat antihuman IgG antibody (Catalogue number 074–1002, KPL-SeraCare), diluted 1:10,000 in wash buffer, was added to the plates and incubated for 1 h at room temperature. After 3 washes the plates were developed with o-phenylenediamine dihydrochloride (OPD) substrate (Sigma) for 10 min and read on an Infinite® 200 PRO microplate reader (TECAN) at 492 nm. The results were expressed as the ratio of test OD to the OD of the plate negative control; samples with OD ratios > 2 were considered positive for SARS-CoV-2 IgG and those with OD ratio ≤ 2, negative.

To confirm the reproducibility and specificity of the KWTRP ELISA before performing the comparison, we re-tested 467 pre-pandemic samples in the validation set reported in 2020 using the reported procedure [3].

### 3.2 Sample sets and ethical considerations

The characteristics of the test sample populations are summarized in Table 2. The gold-standard negative ‘pre-pandemic’ serum/plasma panels comprised: (1) sera from 327 adult blood donors collected in 2018 as part of research into the quality of transfused blood in coastal Kenya and (2) 189 sera samples from annual cross-sectional surveys for malaria surveillance in coastal Kenya collected between April-May 2018. The gold-standard positive ‘pandemic’ plasma panel comprised plasma from 149 COVID-19 patients sampled ≥7 days after their PCR-positive diagnosis. In addition, we tested a pandemic test panel consisting of serum/plasma from COVID-19 PCR testing (*n* = 676) and samples from early in the pandemic (COVID-19 wave 1, *n* = 176) when SARS-CoV-2 antibody seroprevalence was expected to be low and later when higher SARS-CoV-2 antibody seroprevalence was expected (COVID-19 wave 4, *n* = 176).

**Table 2:** Characteristics of the test sample populations.

Population	Date	Location	Patient group	Designation	Reference
Adult blood donors	2018	Coastal Kenya	Adults investigated for blood transfusion safety	Gold-standard negatives	[3]
Adult cross-sectional survey	2018	Coastal Kenya	Adults investigated in the annual malaria cross-sectional survey	Gold-standard negatives	[3]
SARS-CoV-2 PCR positive cohort	2020	Nairobi	Adults with SARS-CoV-2 PCR positive result	Gold-standard positives	[3]
COVID-19 diagnostic testing patients	2020	Coastal Kenya	Adults and children investigated for SARS-CoV-2 infection	Unknowns	[9]
Adult blood donors during COVID-19 wave 1	May 2020	Countrywide	Adults investigated for blood transfusion safety	Unknowns	[10, 11]
Adult blood donors during COVID-19 wave 4	August 2021	Countrywide	Adults investigated for blood transfusion safety	Unknowns	[10]

Commercial negative and positive control calibrators were supplied by the manufacturer for WANTAI. For the KWTRP ELISA the positive control was monoclonal antibody (CR3022) and a pool of pre-pandemic sera from 50 Kenyan adults was used as negative control.

This study was approved by the Scientific and Ethics Review Unit (SERU) of the Kenya Medical Research Institute (Protocol SSC 3426). Before the blood draw, donors gave individual consent the use of their samples for research. Ethical approval was obtained for collection, storage and further use for the sample sets used in the validation assays (SERU numbers: 1433, 3149, 3426).

### 3.3 Statistical analyses

Data analysis was conducted using GraphPad Prism v9, R v4.1.0 and Stata 15.0. Percentage agreement, specificity, sensitivity and prevalence were calculated for both assays using the same sample sets. Pairwise comparisons between the WANTAI and KWTRP were done using the McNemar's test. To explore whether differences between the two assays are intrinsic to the assay or simply a result of the selected cut-off for each assay, we plotted ROC curves for both assays using the positive and negative gold standard panels as test samples and calculated their area under the curves. Finally, we assessed the assays’ reproducibility by examining the raw ODs and coefficient of variation (CV) for the negative and positive controls for all the test runs.

## 4 Results

**Fig. 1:**
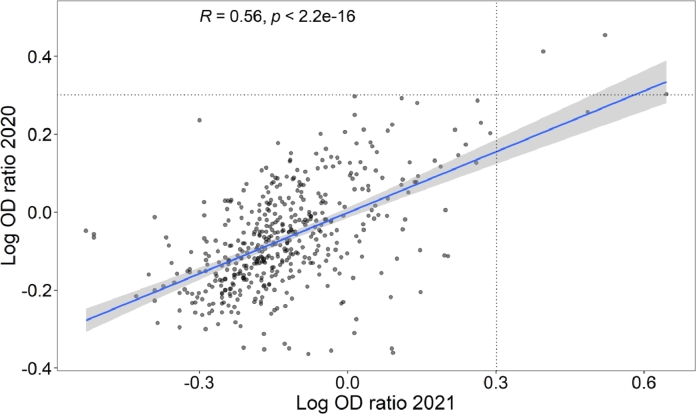
Distribution of OD ratios of the KWTRP ELISA in 2020 and in 2021 using 467 gold standard negatives as a test population. The cut-off for positivity is shown with the dotted lines.

In the intra-assay comparison, the KWTRP ELISA was highly reproducible with specificity of 0.99 (95% CI, 0.98–1.00) in both 2020 and 2021 ELISA. Among the 467 true negative samples both assays classified 3 (0.64%) samples as false positives and the 2021 ELISA classified an additional 1 (0.21%) as false positive (Fig. 1). There was positive correlation between the 2020 OD ratios and those of 2021 ELISA (Fig. 1). Because of minimal changes in the assay performance, WANTAI results were compared to KWTRP results already obtained in 2020.

In the inter-assay comparison, both assays showed very high specificity and sensitivity (Table 3). WANTAI specificity was slightly lower than KWTRP specificity using both negative sample sets, the 2018 pre-pandemic coastal Kenya blood donors and the 2018 malaria cross-sectional survey samples. By contrast, WANTAI showed a slightly higher sensitivity than KWTRP using the 149-gold standard positive samples. Among the three sets of unknown samples, COVID-19 diagnostic testing patients, blood donors during COVID-19 wave 1 and blood donors during COVID-19 wave 4, the prevalence was 0.179, 0.023 and 0.830, respectively, for WANTAI and 0.152, 0.011 and 0.773, respectively, for KWTRP (Table 3).

**Table 3:** Test performance characteristics and estimated prevalence of WANTAI and KWTRP ELISAs for SARS-CoV-2 antibodies in different test populations.

Test population		WANTAI		KWTRP	
	N	Negative	Specificity	Negative	Specificity
**Pre-pandemic samples (gold standard negative)**					
Adults, coastal Kenya blood donors, 2018	327	320	0.979	326	0.997
Adults, cross-sectional survey, 2018	189	187	0.989	189	1.000
	**N**	**Positive**	**Sensitivity**	**Positive**	**Sensitivity**
**Pandemic positives (gold standard positive)**					
SARS-CoV-2 PCR positive cohort	149	142	0.953	138	0.926
	**N**	**Positive**	**Prevalence**	**Positive**	**Prevalence**
**Test samples (unknowns)**					
COVID-19 diagnostic testing patients	676	121	0.179	103	0.152
Adults, Kenya blood donors COVID-19 wave 1	176	4	0.023	2	0.011
Adults, Kenya blood donors COVID-19 wave 4	176	146	0.830	136	0.773

Results of a pairwise comparison showed there was very high overall agreement between the assays (Table 4). However, WANTAI resulted in more false positives than KWTRP in the pre-pandemic samples, the 2018 coastal Kenya blood donors and 2018 malaria cross-sectional survey samples. Both assays classified 4.7% of the positive samples as false negatives. Generally, WANTAI resulted in more positive results than KWTRP in the pandemic test panel reflecting its slightly higher sensitivity and slightly lower specificity compared to KWTRP.

**Table 4:** Pairwise comparison of WANTAI and KWTRP ELISA on different sample sets.

			KWTRP		
Sample type	N	WANTAI	Pos	Neg	P-value*
**Pre-pandemic samples (gold standard negative)**					
Adults, coastal Kenya blood donors, 2018	**327**	**Pos**	0 (0.000)	7 (0.047)	0.07
		**Neg**	1 (0.003)	319 (0.976)	
Adults, cross-sectional survey, 2018	**189**	**Pos**	0 (0.000)	2 (0.011)	0.5
		**Neg**	0 (0.000)	187 (0.989)	
**Pandemic positives (gold standard positive)**					
SARS-CoV-2 PCR positive cohort	**149**	**Pos**	138 (0.926)	4 (0.027)	0.12
		**Neg**	0 (0.000)	7 (0.047)	
**Test samples (unknowns)**					
COVID-19 testing samples	**676**	**Pos**	77 (0.114)	44 (0.065)	0.04
		**Neg**	26 (0.039)	529 (0.783)	
Adults, Kenya blood donors COVID-19 wave 1	**176**	**Pos**	1 (0.006)	3 (0.017)	0.6
		**Neg**	1 (0.006)	171 (0.972)	
Adults, Kenya blood donors COVID-19 wave 4	**176**	**Pos**	132 (0.750)	14 (0.0795)	0.03
		**Neg**	4 (0.023)	26 (0.148)	

⁎ McNemar's Chi square.

In a comparison of ROC curves for the WANTAI and KWTRP assays using all the gold standard positive and negative samples, the WANTAI curve was marginally closer to the optimal point at the top left of the plot with AUC of 0.9841 slightly higher than the KWTRP AUC of 0.9726.

Performance of both ELISAs, examined by coefficient of variation of negative and positive controls, was as expected with little inter-assay variation (Fig. 3). The WANTAI and KWTRP positive control CV were 15.7% and 2.64% while negative control CV were 23.3% and 8.75% respectively (Fig. 3).

## 5 Discussion

**Fig. 2:**
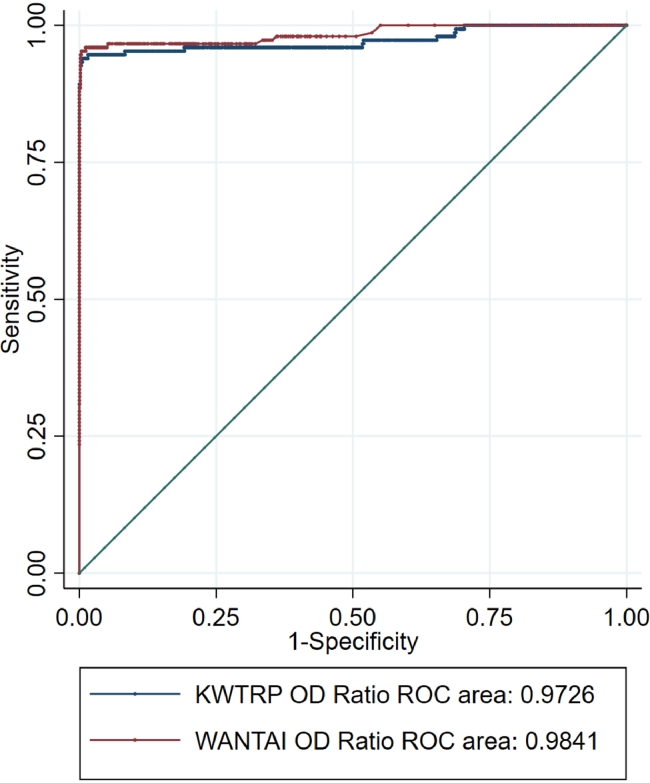
ROC curves for WANTAI and KWTRP ELISAs using all gold standard positives and all gold standard negatives as a test population.

We compared the two ELISAs using the same sample sets. Overall, at the standard cut-offs, the KWTRP assay showed slightly higher specificity than the WANTAI assay which was in turn slightly more sensitive. This was evident in the higher number of positive samples detected by WANTAI in all the sample sets including the positive gold standard panel and false positives in the pre-pandemic sample set. The false positives in the pre-pandemic sample set implies that at low prevalence, the WANTAI assay is likely to overestimate population seroprevalence, transmission and cumulative infection compared to the KWTRP assay and therefore less accurate. By contrast, at high prevalence, WANTAI assay is likely to detect more true positives than KWTRP and therefore more accurate. This trade-off between sensitivity and specificity illustrates that the differences in performance between the assays reported in Table 3 are partly attributable to differences in threshold selection along the ROC curves (Fig. 2). Nonetheless, the additional positives observed with WANTAI could also be contributed by other immunoglobulin classes such as IgA and IgM which are detected by WANTAI but not KWTRP. There were some samples in the pandemic test panel that were positive by KWTRP but negative by WANTAI, which was unexpected because of the slightly higher sensitivity by WANTAI. However, since WANTAI is based on the receptor binding domain while KWTRP on the whole spike protein, the latter contains additional epitopes that could drive responses not observed by the former [12].

**Fig. 3:**
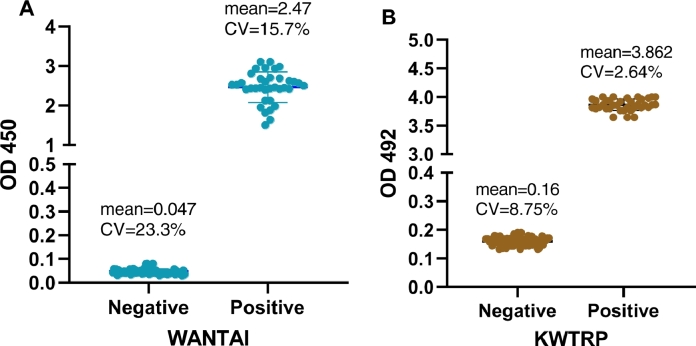
Reproducibility of the WANTAI (A) and KWTRP (B) ELISAs by examining the raw ODs and coefficient of variation for the negative and positive controls for all the test runs done during the comparison. WANTAI negative control ODs were expected to be ≤ 0.100 and ≥1 for positive controls while the KWTRP negative control ODs were expected to be < 0.2 and >3 for positive controls. Performance of both ELISAs was as expected with little inter-assay variation.

The sensitivity of WANTAI observed in this study was comparable to its reported sensitivity, 95.3% compared to 94.4%, while specificity was slightly lower but still comparable in the two sample sets 97.9% and 98.4% compared to the reported 100% [8]. Comparable high sensitivities and specificities have been reported elsewhere for the WANTAI assay [6, 8, 13]. Although the sample numbers and sets (for WANTAI) were different from the ones used to define the reported sensitivities and specificities, finding comparable values inspire confidence in both assays, as they also showed high degrees of reproducibility (CV = <30%) illustrated by the raw OD values of their positive and negative controls.

Overall, both assays showed excellent characteristics to discriminate SARS-CoV-2 seropositive and seronegative individuals and estimate seroprevalence accurately across a wide range of seroprevalences.

## 6 Funding

This research was funded in whole or in part by the Wellcome Trust (grants 220,991/Z/20/Z and 203,077/Z/16/Z). JAGS is funded by a Wellcome Trust Fellowship (214,320). For the purpose of Open Access, the author has applied a CC-BY public copyright license to any author accepted manuscript version arising from this submission.

## CRediT authorship contribution statement

**James Nyagwange:** Conceptualization, Methodology, Investigation, Formal analysis, Writing – original draft, Writing – review & editing. **Bernadette Kutima:** Investigation, Formal analysis, Writing – review & editing. **Kennedy Mwai:** Formal analysis, Writing – review & editing. **Henry K. Karanja:** Investigation, Writing – review & editing. **John N. Gitonga:** Investigation, Writing – review & editing. **Daisy Mugo:** Investigation, Writing – review & editing. **Sophie Uyoga:** Investigation, Writing – review & editing. **James Tuju:** Investigation, Writing – review & editing. **Lynette I. Ochola-Oyier:** Investigation, Writing – review & editing. **Francis Ndungu:** Investigation, Writing – review & editing. **Philip Bejon:** Writing – original draft, Writing – review & editing. **Ambrose Agweyu:** Conceptualization, Methodology, Writing – review & editing. **Ifedayo M.O. Adetifa:** Conceptualization, Methodology, Writing – original draft, Writing – review & editing. **J.Anthony G. Scott:** Conceptualization, Methodology, Formal analysis, Writing – original draft, Writing – review & editing. **George M. Warimwe:** Conceptualization, Methodology, Formal analysis, Resources, Funding acquisition, Writing – review & editing.

## Declaration of Competing Interest

The authors declare that they have no known competing financial interests or personal relationships that could have appeared to influence the work reported in this paper.
